# Maternal Nutritional Status and Pregnancy Outcomes Post-bariatric Surgery

**DOI:** 10.1007/s11695-021-05822-y

**Published:** 2022-02-15

**Authors:** Sara H. Alamri, Ghalia N. Abdeen

**Affiliations:** 1 Department of Community Health Science, Clinical Nutrition, College of Applied Medical Sciences, King Saud University, Riyadh, Saudi Arabia; 2 Department of Clinical Nutrition Services, King Abdulaziz Medical City, King Saud bin Abdulaziz University for Health Sciences, King Abdullah International Medical Research Center, Riyadh, Saudi Arabia; 3 Strategic Center for Diabetes Research, College of Medicine, King Saud University, Riyadh, Saudi Arabia

**Keywords:** Obesity, BMI, Pregnancy outcomes, Neonatal outcomes, Post-bariatric surgeries, Maternal nutritional status, Pregnancy complications

## Abstract

Obesity in childbearing women leads to pregnancy-related complications such as gestational diabetes mellitus, pregnancy-associated hypertensive disorders, and macrosomia. Weight loss helps reduce these complications. Studies show bariatric surgery reduces obesity-related complications during and after pregnancy. However, bariatric surgery might be associated with adverse outcomes, such as low birth weight and small-for-gestational-age infants. In addition, several studies suggest pregnancy occurring less than a year post-bariatric surgery adversely affects pregnancy outcomes and causes micronutrients deficiency since the dramatic weight loss occurs in the first year. These adverse outcomes may lead to nutritional malabsorption, such as anemia and low vitamin B12 and folic acid levels. The review aims to overview obesity-related complications during pregnancy and the benefits and risks of bariatric surgery on pregnancy outcomes and maternal nutrition status.

**Graphical abstract:**

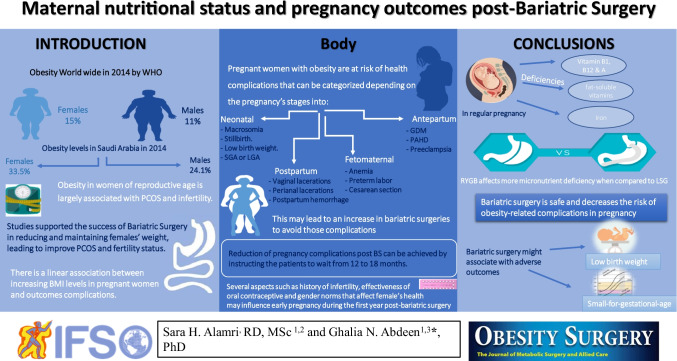

## Introduction

Obesity is a global epidemic, and it has tripled from 1975 to 2016 worldwide. In 2016, 11% men and 15% women were obese [1]. A higher incidence of obesity in females is also reflected in Saudi society where prevalence is 33.5% among females compared to 24.1% among males [2].

With obesity, other complications such as type 2 diabetes mellitus (T2DM), hypertension, and hyperlipidemia occur, but obesity in women of childbearing age poses added complications such as polycystic ovarian syndrome (PCOS) and infertility [3–5]. Furthermore, pregnant women with obesity are at higher risk of pregnancy-associated hypertensive disorders (PAHD) and gestational diabetes mellitus (GDM). Also, maternal and neonatal complications associated with obesity include preterm labor, macrosomia, and stillbirth [6, 7]. Consequently, weight reduction reduces these complications, increases the pregnancy rate, and reduces obesity complications in general for both neonates and mothers [8, 9].

One of the most effective weight loss and long-term weight management methods is bariatric surgery; it is shown to be safe and successful in reducing obesity comorbidities and improving the quality of life [5, 10]. Furthermore, studies show the benefits of bariatric surgery in reducing the complications related to obesity that may affect pregnancy and neonatal outcomes [11–13]. For example, it might lead to malabsorption in micronutrients vital for fetal development during pregnancy [14–16].

This review gives a comprehensive view of obesity-related complications during pregnancy, in addition to the benefits and risks of bariatric surgery on pregnancy outcomes and maternal nutrition status.

## Obesity

Obesity is the accumulation of adipose tissue in the body. Numerous factors contributing to increasing obesity prevalence in Saudi Arabia could be genetic, behavioral, and environmental factors; higher calorie intake with a sedentary lifestyle have exacerbated the condition [5, 17]. 

### Prevalence of Obesity in Saudi Arabia

It is well known that obesity prevalence has increased dramatically over the last decade worldwide. Moreover, obesity has increased rapidly in Saudi Arabia from 1990 to 2005, from 22.1% to 35.6% [18]. A study conducted in Saudi Arabia, including > 17,000 participants, indicated that obesity increased 44% in females compared to 26.4% in males, with a higher prevalence among females in the 40 to 49 years age group [19].

Another study conducted on more than 10,000 participants showed higher obesity and morbid obesity prevalence in Saudi Arabia among females (33.5%, 4.7%) compared to males (24.1%, 2.5%), respectively [2]. The future projection in obesity prevalence for females in Saudi Arabia was estimated to rise from 21% in 1992 to 78% in 2022 [18]. This gender disparity of obesity in the Middle East especially in Saudi Arabia is exacerbated among women.

Despite the biological factors that may contribute to these gender variations such as metabolism, menopause, and adiposity distribution, there are several sociodemographic and culture factors [20], for instance, the early marital age of females. A demographic survey from 2016 showed that the number of Saudi women married from 15 to 19 years was around 24,000, which can lead to unregulated multiple pregnancies as those women had around 12,000 live-born children [21]. Weight retention after each pregnancy might lead to long-term weight gain [22]. However, with increasing awareness and education, there have been changes in the last decade [23].

### Obesity-related Complications

Obesity is associated with comorbidities such as T2DM, hypertension, cancer, cardiovascular diseases (CVDs), infertility, and PCOS [2, 5]. Obesity in women of reproductive age is associated mainly with PCOS and infertility. The polycystic ovarian syndrome is defined as an endocrine condition that occurs commonly in females of childbearing age [3, 8]. The prevalence of PCOS in Saudi Arabia remains unknown. However, a prospective study including 83 Saudi females with PCOS shows that 51% were obese [24]. 

In a cross-sectional clinical study conducted on 201 female students (mean age 21.3 ± 2.1), 53.7% of the participants were diagnosed with PCOS using ultrasound in the University Medical Center in Saudi Arabia [25]. The high prevalence of PCOS in this study was attributed to the higher prevalence of obesity in Saudi Arabia. However, the weight of the participants was not measured in the study. Another cross-sectional study conducted among female university students using a self-reporting questionnaire on PCOS reported that 16% of participants had PCOS and 28% of the PCOS participants were overweight and obese [26]. Despite the small sample size in these three studies, they all highlighted the association between obesity and PCOS [24–26].

### Obesity-related Complications in Pregnancy

Pregnant women with obesity are at risk of health complications and are categorized according to four stages in pregnancy: antepartum, fetomaternal, postpartum, and neonatal [7].

#### Antepartum Outcomes

Antepartum is defined as the period during pregnancy and before the onset of delivery [27]. Studies conducted in Saudi Arabia showed the effects of obesity on antepartum outcomes such as PAHD, GDM, and preeclampsia [7, 28]. These outcomes were higher among pregnant women with obesity than those with normal weight or underweight (Table 1). A prospective cohort conducted in France and Canada also showed a higher percent of PAHD among morbidly obese patients than with other BMI groups [29].

**Table 1:** Antepartum outcomes

Study	PAHD				GDM				Preeclampsia			
	UWT	NW	O/W	Obesity	UWT	NW	O/W	Obesity	UWT	NW	O/W	Obesity
El-Gilany and Hammad (2010) [28]	0	4%	4.9%	6.1%	0	3%	4.4%	8.6%	0	3%	3.8%	5.9%
Fallatah et al. (2019) [7]	NW	Obese I	Obese II	MO	NW	Obese I	Obese II	MO	NW	Obese I	Obese II	MO
	2.5%	2.58%	0.38%	1.94%	0.03%	12.26%	15.27%	18.71%	2.5%	2.09%	0.76%	2.58%
Sun et al. (2020) [30]	UWT	NW	O/W	Obesity	UWT	NW	O/W	Obesity	UWT	NW	O/W	Obesity
	1.2%	2.0%	6.5%	11.9%	7.9%	11.0%	19.0%	20.3%	-	-	-	-

In Fallatah et al.’s (2019) study, most of the pregnant participants were classified as obese class 1

*GDM*, gestational diabetes mellitus; *PAHD*, pregnancy-associated hypertensive disorders;

*UWT*, underweight; *NW*, normal weight, *O*/*W*, overweight; *MO*, morbid obesity

#### Fetomaternal Outcomes

Fetomaternal outcomes are referred to outcomes that contribute to both fetus and mother, for example, cesarean section delivery, preterm labor, and anemia [7]. A Chinese cohort studied the effects of pre-pregnancy BMI on maternal and fetal outcomes in over 3000 pregnant participants. It observed a higher percentage of cesarean sections among pregnant women with overweight and obesity when compared to normal and underweight participants [30]. However, there were no details on whether cesarean section deliveries were planned or unplanned or whether the pregnancy was nulliparous or parous with repeated cesarean delivery. Similarly, a systematic review found a correlation between an increased rate of unplanned cesarean sections and increased BMI [6].

Several studies concluded that the incidence of cesarean section, preterm labor, and anemia was higher in pregnant women with obesity when compared to underweight and normal body weight (Table 2). Two Saudi studies evaluated the relation between anemia with BMI as one of their study variables. Fallatah et al. (2019) found a higher percentage of anemia among participants with morbid obesity (61.3%) [7]. In contrast, the other study reported a higher percentage of anemia among underweight participants (49.2%) [28]. Anemia was found to be moderate or severe during the first trimester [31]. Two studies observed that preterm labor was associated with a BMI of ≥ 30 kg/m^2^ [32, 33]. While in another study, it was associated with BMI < 18 kg/m^2^ [28].

**Table 2:** Fetomaternal outcomes

*Study*	*Cesarean section delivery*								*Preterm labor*						*Anemia*					
	NW		Obese I		Obese II		MO		NW	Obese I	Obese II		MO		NW	Obese I	Obese II		MO	
Fallatah et al. (2019) [7]	15%		63.9%		79%		79.4%		7.1%	11.9%	11.83%		12.66%		-	58.9%	59.5%		61.3%	
Schummers et al. (2015) [32]	< 18.5	25– < 30		30– < 35		35– < 40		BMI ≥ 40	< 18.5	25– < 30	30– < 35	35– < 40		BMI ≥ 40	< 18.5	25– < 30	30– < 35	35– < 40		≥ 40
	21.8%	33.1%		38.2%		43.1%		49.7%	9.2%	7.5%	8.4%	8.8%		10.3%	-	-	-	-		-
Zhao et al. (2019) [33]	18.5 ≥ 22.9	≥ 24–24.9		≥ 25–27.9		≥ 28–29.9		BMI ≥ 30	18.5 ≥ 22.9	≥ 24–24.9	≥ 25–27.9	≥ 28–29.9		BMI ≥ 30	18.5 ≥ 22.9	≥ 24–24.9	≥ 25–27.9	≥ 28–29.9		BMI ≥ 30
	21.5%	28.6%		28.3%		33.5%		32.8%	5%	5.4%	7%	8.7%		15.3%	2.8%	2.7%	2.7%	2.2%		2.3%

In Zhao et al.’s (2019) study, there was no correlation between anemia and pregnant obese participants

*UWT*, underweight; *NW*, normal weight; *O*/*W*, overweight; *MO*, morbid obesity

#### Postpartum Outcomes

The developed outcomes after delivery are known as postpartum outcomes. Studies show that some of these outcomes increase with increasing BMI during pregnancy, including postpartum hemorrhage and vaginal and perianal lacerations [7, 33, 34]. A study with 300 pregnant with obesity and 300 non-obese pregnant as a control group showed greater postpartum hemorrhage among the pregnant in the obesity group compared to the control group [35]. In contrast, a systematic review reported no increase in postpartum hemorrhage with BMI increase [6].

A prospective study from two cohorts shows overweight pregnant women have an increased risk of perianal lacerations [34]. Similarly, another study illustrated a relation between vaginal and perianal lacerations with obesity caused by the increase of macrosomia among pregnant women with obesity [7]. Contrary, a recent systematic review shows fewer perianal lacerations associated with increased BMI among postpartum subjects, attributed to the higher number of cesarean section deliveries, as mentioned previously, as fetomaternal outcomes [6]. Studies that found a relation between postpartum hemorrhage, vaginal lacerations, perianal lacerations, and higher BMI sample groups are shown in Table 3.

**Table 3:** Postpartum outcomes

Study	Postpartum hemorrhage						Vaginal lacerations						Perianal lacerations					
	NW	Obese I		Obese II		MO	NW	Obese I		Obese II		MO	NW	Obese I		Obese II		MO
Fallatah et al. (2019) [7]	1%	1.3%		0.8%		1.3%	-	7.4%		3.4%		3.2%	-	4.7%		1.5%		2.6%
Haseeb (2017) [35]	Non-obese			Obese			Non-obese			Obese			Non-obese			Obese		
	2%			5.1%			-			-			-			-		
Rahman et al. (2020) [34]	OR (95% CI)						OR (95% CI)						OR (95% CI)					
	UWT		NW		O/W		UWT		NW		O/W		UWT		NW		O/W	
	1.11 (0.77–1.61)		1		1.68 (1.12–2.53)		-		-		-		1.00 (0.64–1.56)		1		2.46 (1.54–3.92)	

*UWT*, underweight; *NW*, normal weight; *O*/*W*, overweight; *MO*, morbid obesity

#### Neonatal Outcomes

Outcomes related to the fetus are known as neonatal outcomes. Neonates born to mothers with obesity have adverse outcomes such as macrosomia, stillbirth, low birth weight, and small-for-gestational age (SGA) or large-for-gestational age (LGA) [6]. Macrosomia is defined as birth weight > 4000 g, while low birth weight is < 2500 g; SGA is defined as < 10th percentile, and LGA as > 90th percentile [6, 33].

Pregnant women with high BMI have a higher risk of macrosomia, stillbirth, and other neonatal outcomes [6]. A Chinese study with a sample size of over 11,000 found increased pre-pregnancy BMI linked to macrosomia and LGA, while SGA infants were linked to decreased pre-pregnancy BMI. On the other hand, there was no correlation between low birth weight and pre-pregnancy BMI [33]. Similarly, in another study, higher pre-pregnancy BMI was associated with a higher incidence of LGA and lower SGA [36]. Low birth weight was associated with underweight subjects, while macrosomia and stillbirth increased among higher BMI subjects shown in Table 4. Whereas SGA was higher among underweight subjects, and LGA was associated with higher BMI subjects, as shown in Table 5.

**Table 4:** Neonatal outcomes

Study		Birth weight					Macrosomia							Stillbirth					
	UWT	NW		O/W		Obese	UWT	NW			O/W	Obese		UWT	NW		O/W		Obese
El-Gilany and Hammad (2010) [28]	26.90%	11.70%		9.60%		5.80%	0	0.70%			2.10%	4.40%		1.50%	0.70%		1.10%		1.10%
Schummers et al. (2015) [32]	< 18.5	25– < 30	30– < 35	35– < 40	BMI ≥ 40		< 18.5	25– < 30	30– < 35	35– < 40	BMI ≥ 40			< 18.5	25– < 30		30– < 35	35– < 40	BMI ≥ 40
	3187 ± 524	3505 ± 565	3548 ± 595	3572 ± 611	3619 ± 623		0.40%	2.80%	3.80%	4.50%	6.10%			0.30%	0.30%		0.40%	0.40%	0.60%
Fuchs et al. (2017) [29] (French cohort)	UWT	NW		Obese			UWT	NW	O/W		Obese			UWT	NW	O/W	Obese		
				Obese I	Obese II	MO					Class I	Class II	MO				Class I	Class II	MO
	3082 ± 662	3181 ± 687	3226 ± 741	3180 ± 828	3184 ± 804	3157 ± 842	4.30%	6.60%	9.20%		10%	9%	9.10%	0.30%	0.50%	0.70%	0.80%	0.90%	

*UWT*, underweight; *NW*, normal weight; *O*/*W*, overweight; *MO*, morbid obesity

**Table 5:** Neonatal outcomes

Study	SGA											LGA								
	UWT			NW			O/W			Obesity		UWT		NW		O/W			Obesity	
Enomoto et al. (2016) [36]	13.21%			8.67%			7.24%			7.06%		5.46%		10.05%		17.36%			22.60%	
Zhao et al. (2019) [33]	< 18 kg/m^2^	18.5 ≥ 22.9	≥ 23–23.9		≥ 24–24.9			≥ 25–27.9	≥ 28–29.9		BMI ≥ 30	< 18 kg/m^2^	18.5 ≥ 22.9	≥ 23–23.9	≥ 24–24.9		≥ 25–27.9	≥ 28–29.9		BMI ≥ 30
	3.70%	2.40%	1.80%		2.20%			2.00%	1.70%		2.80%	16.40%	24.20%	30%	32.40%		31.70%	35.70%		34.50%
Sun et al. (2020) [30]	UWT		NW			O/W				Obesity		UWT		NW		O/W			Obesity	
	7.60%		%6.00			3.20%				0		6.00%		9.60%		19.20%			18.60%	

*UWT*, underweight; *NW*, normal weight; *O*/*W*, overweight; *MO*, morbid obesity

A linear association between increasing BMI in pregnant women and outcomes complications may increase bariatric surgeries to avoid those complications [14]. This might be a challenge for obstetricians and bariatric surgeons, as more studies are needed to identify different outcomes in post-bariatric surgery pregnancies.

### Obesity Treatment Modalities

Obesity can be managed by non-surgical treatments, including dietary, psychological, and behavioral interventions, besides FDA-approved medical treatments for weight loss [5, 37]. Anti-obesity medications such as phentermine, orlistat, and liraglutide are recommended as an adjunct to lifestyle interventions [5]. In a randomized controlled trial, Wadden et al. (2018) evaluated whether intensive behavioral therapy (IBT) would increase weight loss further when accompanied with liraglutide. At week 24, the IBT plus liraglutide group lost more weight than the IBT alone group (12.2 ± 0.6%, 10.1 ± 0.6%), respectively, which was significantly different and showed the importance of adjunctive therapies in chronic weight management [37].

If obesity cannot be managed by non-surgical treatment, long-term weight management with surgical treatment options might be considered when there is no significant weight loss or no improvement in comorbidities is seen [5].

Several studies have supported bariatric surgery in reducing and maintaining females’ weight, leading to improved PCOS and fertility status [3, 4, 8]. Weight reduction has shown improvement not only in PCOS and fertility conditions but also in pregnancy outcomes and complications. For instance, a study with PCOS patients who underwent vertical sleeve gastrectomy had more significant weight loss, and 22% became pregnant compared to 3% in non-PCOS patients [8]. In addition, a retrospective study of 44 patients with PCOS who underwent bariatric surgery showed a significant reduction in body mass index (BMI), improvement in PCOS, irregular menses, and marginally elevated ovarian volume compared with baseline pre-surgery [4].

In the same context, a systematic review and meta-analysis evaluated all types of bariatric surgeries performed to treat patients with PCOS and infertility [3]. The mean BMI of 2130 female participants pre-surgery was 46.3 kg/m^2^, compared to 34.2 kg/m^2^ post-surgery, and the incidence of PCOS pre-surgery significantly dropped to 7.1%, from 45.6% a year after surgery. Moreover, the incidence of infertility pre-surgery significantly reduced from 18.2 to 4.3% a year post-surgery [3].

## Bariatric Surgery

One of the most effective methods for weight loss and maintenance is bariatric surgery. The American Society for Metabolic and Bariatric Surgery (ASMBS) defines bariatric surgeries as procedures causing significant weight loss by modifying the digestive system to restrict the nutrients, causing malabsorption or mixing both restriction and malabsorption. Bariatric surgeries have different procedures, with advantages and disadvantages [38]. The most common bariatric surgeries are laparoscopic sleeve gastrectomy (LSG), Roux-en-Y gastric bypass (RYGB), adjustable gastric band (AGB), and biliopancreatic diversion with a duodenal switch (BPD/DS) [5, 39]. Bariatric surgery is also known as metabolic surgery since it encompasses the improvement of other metabolic diseases, including T2DM, hypertension, and hyperlipidemia [40].

### Different Bariatric Surgical Procedures

A survey within 50 nations in the International Federation for the Surgery of Obesity and Metabolic Disorders (IFSO) shows that the most common bariatric surgeries are RYGB (46.6%) and LSG (27.8%), followed by AGB (17.8%) and BPD/DS (2.2%) [41]. An updated survey showed LSG (53.6%) became the most performed bariatric surgery compared with RYGB (30.1%), followed by one anastomosis gastric bypass (OAGB) (4.8%) [39]. 

In 2016, more than 20,000 bariatric surgeries were performed in Saudi Arabia, of which over 16,000 were LSG, 2800 were OAGB, and 800 were RYGB surgeries. Other procedures such as AGB and BPD/DS were less common [39]. RYGB involves creating a small pouch from the upper part of the stomach, which is then attached to the small intestine bypassing a section of the small intestine and limiting the absorbed calories [39, 42]. The OAGB, also known as mini-gastric bypass, differs from the RYGB as it contains two anastomoses rather than one [39].

On the other hand, LSG is a procedure that reduces the stomach size by 85%, creating a tube pouch, restricting the amount of food consumed. As a result, it has a lower complication rate and lacks anastomosis and malabsorption of the nutrients since the intestine is intact [38, 42].

In a recent study, the number of females (*n* = 66) who underwent bariatric surgery was more than males (*n* = 46) [43]; which was similarly reported in another Saudi study showing a higher number of female participants (61.43%) when compared to males (38.7%) [44]. A study by Hult et al. (2019) shows that the main reasons for women to undergo bariatric surgery are seeking weight loss and improving self-esteem [45]. In an online survey, bariatric surgeons reported that one of the occasional reasons to undergo bariatric surgery was to enhance fertility conditions [46].

In a recent Saudi study, the mean age for males and females who underwent bariatric surgery was 36.87 ± 11.44 [44]. Likewise, another study showed the mean age was around 37.4 ± 11.1 [43].

Bariatric surgery is considered when all non-surgical interventions such as dietary, behavioral, and pharmacological treatments have failed to achieve weight loss [5]. Individuals with morbid obesity (BMI ≥ 40 kg/m^2^) or individuals with obesity (BMI ≥ 35 kg/m^2^) with obesity-related comorbidities are the main indications for bariatric surgery [5, 47].

A linear association between increasing BMI in pregnant women and outcomes complications may increase bariatric surgeries to avoid those complications [14]. This might be a challenge for obstetricians and bariatric surgeons as more studies are needed to identify different outcomes in post-bariatric surgery pregnancies.

## Bariatric Surgery and Pregnancy

### Surgery-to-Conception Interval

There are limited data regarding the recommended period to avoid pregnancy post-bariatric surgery and if the timing of conception would possibly affect the pregnancy outcomes or the mother’s nutrition status [47]. Nonetheless, clinical practice guidelines for bariatric surgery patients suggested delaying pregnancy from 12 to 18 months post-surgery. However, those pregnant before the recommended time should be closely monitored to ensure fetal health, appropriate weight gain during pregnancy, and adequate nutritional supplements [48].

An observational study found that women who became pregnant before one-year post-surgery had higher pre-gestational BMI (34.6 ± 7.7 kg/m^2^) and higher incident of stillbirth (35.5%) compared to those who became pregnant after one year (BMI 30.4 ± 5.3 kg/m^2^, stillbirth incidence (16.8%)) [49]. In another study, among 63 pregnancies, 11 (18%) occurred within 12 months, and 52 (83%) after 12 months post-LSG. Women who conceived after 12 months post-LSG had significantly higher weight gain. On the other hand, BMI at conception, low birth weight, and SGA were higher in women who conceived within 12 months post-surgery [50].

However, a French study of pregnancy outcomes among 56 women who underwent RYGB found no relation between the timing of surgery and conception with the outcomes, which might be due to the higher mean time from surgery to pregnancy (32 ± 14 months) [11]. Likewise, no significant difference was found between the two study groups who became pregnant ≤ 18 months and > 18 months regarding preterm labor, birth weight, and SGA or LGA [51].

Another study concluded higher gestational weight gain (GWG) among those who became pregnant around 36 months post-bariatric surgery compared to those who became pregnant around 13 months post-bariatric surgery (median 11 kg versus 8 kg); no significant difference was found in the other fetomaternal outcomes [52].

### Gestational Weight Gain Post-maternal Bariatric Surgery

In regular pregnancies, the Institute of Medicine (IOM) recommended GWG based on the pregnancy BMI, as shown in Table 6. Those who followed these guidelines had better overall pregnancy outcomes than those who did not follow them [53]. However, there is limited data to predict whether those guidelines can be utilized in post-bariatric pregnancies [54].

**Table 6:** Recommendations for total weight gain during pregnancy, by pregnancy BMI

Pregnancy BMI	Total weight gain (kg)
Underweight (BMI < 18 kg/m^2^)	12.5–18
Normal weight (18.5–24.9 kg/m^2^)	11.5–16
Overweight (25.0–29.9 kg/m^2^)	7–11.5
Obese (BMI ≥ 30.0 kg/m^2^)	5–9

A recent study on neonatal outcomes post-maternal bariatric surgery indicated higher infants with SGA among insufficient weight gain mothers, while LGA infants were higher among the excessive weight gain mothers when compared to the other GWG groups. In addition, preterm birth was higher among the insufficient weight gain group [54].

It is well known that weight regain is possible post-bariatric surgery. Therefore, it is important to monitor the weight closely during post-bariatric surgery pregnancy to avoid excessive GWG which can cause postpartum weight retention [55]. Despite the fact of weight trajectory in post-bariatric surgery pregnant tend to gain less weight when compared with those with no history of bariatric surgery [53].

On one hand, for those with a tendency to lose weight, it might lead to negative neonatal outcomes such as SGA and low birth weight [56]. In these cases, patients should be referred to a clinical dietitian to assess their nutritional status and oral intake. Oral supplements can be prescribed to avoid weight loss and to ensure adequate protein intake of at least 60 g per day [57]. On the other hand, for those with a tendency to gain more weight, it leads to weight retention after delivery and consequently weight regain [54].

In light of the above, a prospective study showed among 127 post-bariatric pregnancies 24% had inadequate weight gain while 20% were adequate weight gain. However, the majority (56%) showed excessive weight gain, and they had the highest weight retention weight after delivery [54].

In Saudi Arabia, the information about gestation weight gain or the weight retention postpartum is still limited. Therefore, more studies regarding this area need to be addressed. However, a regular follow-up with the clinical dietitian is advisable to prevent excessive weight gain thus future weight regain [54, 57].

### Bariatric Surgeons’ Awareness of Pregnancy Complications

Surgeons’ awareness of post-bariatric surgery pregnancy complications is critical for the patients’ safety. Awareness can help reduce pregnancy complications by instructing the patients to wait from 12 to 18 months before conception [46]. 

A group of bariatric surgeons participated in a cross-sectional study to assess their knowledge about reproductive health in women following bariatric surgery. Online surveys were distributed to bariatric surgeons, 48 responded; the majority (89.6%) considered discussing reproductive health vital to female patients who undergo bariatric surgery. Moreover, around 64% of the surgeons agreed that 12 months is the minimum time after surgery to avoid pregnancy [46].

### Patients’ Awareness about Surgery-to-Conception Interval and Nutritional Knowledge

A recent Saudi study, including 457 female participants from the eastern province of Saudi Arabia, found that most of the targeted population had poor knowledge (73.1%) [58]. However, 33.3% of the participants were well aware of clinical practice guidelines and the period from bariatric surgery to conception (12–18 months) [48, 58]. Moreover, a retrospective study on 106 females in Saudi Arabia showed that pregnancies happened 21.1 ± 13.5 months from surgery [10].

Regarding nutritional knowledge post-bariatric surgery, a Saudi study found that 58.2% of the participants expressed the need for dietitian follow-up in post-bariatric surgery pregnancy [58]. Similarly, another cross-sectional study assessed the knowledge level of 112 participants scheduled for bariatric surgery. The nutritional knowledge score was 42 out of 85, and their awareness of dietary recommendations was medium (50–75%). There was no significant difference between the level of knowledge and the gender of the participants; however, a relationship between nutritional knowledge and education was seen [43].

### Post-surgery Compliance to Preconception Guidelines

Despite the previous Saudi studies that showed more compliance to the period from surgery to pregnancy. The dietitians’ observation in clinics shows many females become pregnant post-bariatric surgery in less than 12 to 18 months. During this period, the patients are predisposed to rapid weight loss, leading to impaired fetal development [51, 59].

Several aspects influence early pregnancy during the first year post-bariatric surgery, such as a history of infertility, the effectiveness of oral contraceptives, and the gender norms in our Saudi society [23, 46, 59, 60]. A prospective study of 650 women assessed pre-bariatric surgery, and 52 women reported a history of infertility and nulliparous. These 52 women have a higher conception rate post-bariatric surgery, with 33 pregnancies reported, and early pregnancy < 18 months [60].

Nevertheless, for women who do not wish to conceive and use contraception, it is preferred to use long-acting reversible contraception (etonogestrel implants and intrauterine devices) before the surgery; considering the physiological and anatomical changes, post-bariatric surgery brings to decrease drug bioavailability [61]. Postoperatively, the use of long-acting contraceptives (e.g., injection and implants) were considered a safe method when compared to the use of oral contraceptives [62]. A study reported oral contraceptives as the most commonly used method (42.3%) post-bariatric surgery by 111 participants. In comparison, 33.3% reported unintentional pregnancy, and only 9% received contraceptive advice pre-bariatric surgery [63].

In Saudi Arabia, 63% of married women do not use any contraceptive methods, while the most common method was the oral contraceptive [21]. Consequently, the dearth of using other contraceptive methods might lead to early conception post-bariatric surgery. Besides, frequent pregnancies are one of the norms in Arab society; for this reason, reproductive health counseling should be addressed preoperatively [23, 62].

## Maternal Nutrition Status Post-bariatric Surgery

In a normal pregnancy, the requirements of vitamins and minerals, such as folate, iron, vitamins B1, B6, B12, and fat-soluble vitamins are increased. Deficiencies may result if these micronutrients are not consumed adequately [15]. Different types of bariatric surgeries result in different levels of micronutrient deficiencies during pregnancy. For example, a study showed higher gestational anemia among women who underwent bariatric malabsorptive surgeries (e.g., RYGB) when compared to restrictive surgeries (e.g., LSG) [13].

Micronutrient supplementations are usually recommended to prevent deficiency in post-bariatric patients during pregnancy [11, 48, 64]. In a retrospective study of 168 post-bariatric surgery pregnancies, three post-bariatric pregnant women were diagnosed with hyperemesis gravidarum, which increases the risk of malnutrition and the need for oral supplements [49].

### Anemia

Numerous studies showed that post-bariatric, pregnant women are more susceptible to developing anemia due to the increased demand and inadequate intake [13, 15, 65]. For instance, twenty-four women were grouped under the four bariatric surgeries they underwent; 69% developed anemia and underwent malabsorptive bariatric surgery (e.g., RYGB and LSG with duodenojejunal bypass) compared to AGB and LSG [13].

Similarly, an observational study investigated 150 pregnancy outcomes of women who underwent three different bariatric surgeries: LSG, BPD, and RYGB. Among the 150 women, 24.2% developed anemia post-BPD and 15.6% post-RYGB pregnancy [66]. A systematic review recently found an increased risk of anemia, which occurred in 17 to 77% of pregnancies post-bariatric surgery, and the participants had lower ferritin levels. No reduction in folic acid levels was found among post-bariatric surgery pregnant women [15]. Currently, guidelines recommended that post-bariatric patients should be supplemented with iron 45–60 mg elemental iron. Laboratory testing for iron, ferritin, and transferrin at least every 3 months in women planning to get pregnant post-bariatric surgery to avoid the risk of developing anemia during pregnancy [57].

### Vitamin B12

A study showed vitamin B12 levels were measured before, during, and after pregnancy. Lower vitamin B12 levels were observed in all types of post-bariatric surgeries, with no further reduction during pregnancy. Serum albumin levels equally decreased during pregnancy in all groups [66].

A recent retrospective study on 123 women evaluated pregnancy and nutritional outcomes of two different bariatric surgeries: LSG and RYGB. The results showed similar deficiencies between both surgeries when assessed during the second trimester; vitamin B12 deficiency was higher among post-RYGB pregnancies. The study also found post-RYGB pregnant women tended to comply with their multivitamin supplementation than post-LSG pregnant women [67]. In a study, the cord blood of 56 post-RYGB pregnancies was tested, and the results revealed higher levels of vitamin B12 with other micronutrients such as magnesium and vitamin E among neonates born after maternal bariatric surgery [11]. The serum level of vitamin B12 or transcobalamin should be checked in the preconception period at least every 3 months, and supplementation of vitamin B12 (1 mg IM) for 3 months is recommended when planning for pregnancy [57].

### Calcium and Vitamin D

A study that analyzed the micronutrients for mothers post-RYGB and their neonates reported lower vitamin D and calcium levels among the mothers at delivery, while in neonates they observed lower calcium levels when compared to the control group. Additionally, vitamin D deficiency was associated with maternal obesity in this study [11]. A prospective cohort study found that women with vitamin D deficiency were at 14% in the first trimester and 6% at delivery [68]. During the preconception period, supplementations of vitamin D should be given to maintain the concentration of 50 nmol/L (1000 IU) and calcium should be added as needed 1200–1500 mg; both micronutrients should be tested at least every 6 months [57].

### Vitamin A

A recent systematic review reported vitamin A deficiency among 90% of the pregnancies post-bariatric surgery. This deficiency was associated with inadequate GWG [15]. A retrospective study revealed that vitamin A deficiency was among 90% of pregnant women who underwent RYGB, around 86.7% of them developed night blindness. Among pregnant women with anemia, 90.9% were vitamin A deficient [65].

Likewise, Devlieger et al. vitamin A deficiency was found to increase throughout pregnancy with 19% in the first trimester and 58% at delivery [68]. In one study, they found vitamin A deficiency among post-RYGB mothers despite being on vitamin A supplement (1200 μg), although less concentration of vitamin A in the cord blood of post-maternal RYGB neonates when compared to the control group [11].

However, retinol-based vitamin A supplementations are not suitable during pregnancy and lactation due to their teratogenic effect [57, 69]. A systematic review found that most vitamin A deficiency cases were reported in pregnancies post-BPD procedures and resulted in neonatal vision complications and preterm birth [70]. In another systematic review, vitamin A deficiency was not associated with gestational age or birth weight. Yet, vitamin A deficiency was associated with inadequate GWG [15].

Cabano et al. had reported one case of aortic dilatation in a neonate who had severe vitamin A deficiency, the neonate’s mother had undergone BPD 16 years before pregnancy, and no vitamin A supplementations were taken [71]. Although, this association between vitamin A deficiency and aortic dilatation has been described previously in animal models [71, 72]. Another case of neonatal aortic dilatation was reported with relation to low vitamin A in both the mother and which improved after given vitamin A supplementation [72]. During pregnancy, vitamin A serum levels should be checked at least every 3 months. While supplementation should be taken in the form of beta-carotene (5000 IU) [57].

### Thiamine

Thiamine (Vitamin B1) is an essential vitamin that has a major role in multiple metabolic pathways, and its deficiency can lead to severe complications, such as Beriberi and Wernicke encephalopathy [73]. In addition, Wernicke encephalopathy has been reported in one case post-RYGB pregnancy with hyperemesis gravidarum [69, 74]. In a recent study, thiamine deficiency was reported in 25.7% of 105 patients post-LSG, even in those on thiamine supplement and it was more among female patients (81%) [73].

However, the data were limited regarding the percentage of thiamine deficiency in pregnant women who have undergone bariatric surgery. Nonetheless, a retrospective study analyzed thiamine levels in women before and after bariatric surgery. The result showed thiamine deficiency was not detected pre-surgery. While 42 women post-bariatric surgery out of 456 had thiamine deficiency. Also, 36 pregnant women who underwent bariatric surgery had thiamine deficiency [75].

Although, in normal pregnancy, concentrations of thiamine decrease throughout pregnancy; however, there are factors that increase thiamine deficiency post-bariatric surgery such as rapid weight loss, insufficient oral intake, and persistent vomiting [68]. During pregnancy, thiamine supplementation 300 mg with vitamin B complex 3 times daily is recommended for pregnant women with vomiting. In case of prolonged vomiting, an intravenous route should be considered [57].

### Other Micronutrients

A systematic review reported a deficiency in several micronutrients, such as vitamin B6, C, K, selenium, and phosphorus during pregnancy post-bariatric surgeries [15]. In addition, if folic acid (folate) deficiency also occurs during pregnancy, this might lead to complications such as neural tube defects in the fetus and megaloblastic anemia [69]. During preconception, folic acid should be taken daily (0.4 mg), and to continue throughout the first trimester, this dose might increase to 4–5 mg in women with obesity or diabetes [57]. Also, selenium deficiency is suggested to be one of the factors of early miscarriages, and it has a role in fetus development. Contrary, too much selenium may contribute to nervous system damage [55].

To prevent deficiencies, the serum indices have to be monitored during pregnancy. Therefore, the serum concentration of full blood count, folic acid, and other micronutrients mentioned above should be tested every 3 months. Also, protein, albumin, phosphate, magnesium, renal, and liver functions should be tested every 6 months. Other extra serum indices must be checked especially during the first trimester, such as zinc, copper, selenium, and vitamin E [57].

## Effects of Bariatric Surgery on Maternal Outcomes

Several studies have proven the benefit of bariatric surgery in improving the maternal and neonatal outcomes associated with obesity, while other studies illustrate the adverse effect of bariatric surgery on some obstetrics outcomes [10, 13, 35, 67]. The differentiation between complications in pregnant women with obesity and post-bariatric surgery pregnancy is shown in Table 7. Each bariatric surgery has a different effect on nutritional absorption, causing some nutritional deficiencies affecting perinatal outcomes, thus emphasizing the importance of studying the different surgeries and their effect on perinatal and maternal outcomes [14, 15].

**Table 7:** The differentiation between complications in pregnant women with obesity and post-bariatric surgery pregnancy

Stages of pregnancy	Complications associated with obese pregnant	Complications associated with post-bariatric surgery pregnant
1st trimester	Vomiting [55] Increase risk of miscarriage [55] Anemia [31]	Vitamin K deficiencies [76] Vitamin D deficiency [68] Vomiting [55] Anemia [68]
2nd trimester	Gestational diabetes mellitus [77] Pregnancy-associated hypertensive disorders [78]	Vitamin B12 deficiency [68]
3rd trimester	Preeclampsia [78]	Anemia [13] Calcium deficiency [76] Increases risk for osteoporosis [76]
Postpartum	Postpartum hemorrhage [7, 33] Postnatal depression [55] Vaginal lacerations [7] Perianal lacerations [7, 33]	Thiamine deficiency [68] Vitamin A deficiency [68]

González et al. (2017) reported that only 5 (3%) out of 168 post-bariatric surgery patients had GDM during their pregnancies, and none had PAHD [47]. Similarly, an observational study investigating four different bariatric surgeries on 24 pregnant women found that only two post-LSG patients with no prior diabetic history developed GDM. Furthermore, the study also found that 3 out of 6 post-AGB patients developed PAHD [49].

Only one GDM case was reported in one study with 56 post-RYGB pregnant women [11]. Although a cohort control study of 596 post-bariatric surgery pregnancies and 2356 matching control of pregnant women with obesity, matched in pre-surgery weight and excluding prior diabetes history, found lower GDM among post-bariatric surgery pregnancies (1.9%) than in the matching control group (6.8%) [12].

A case of dumping syndrome (DS) was reported in post-bariatric pregnant women that usually occurs within one hour of food consumption [79]. This syndrome is attributed to rapid gastric emptying led to symptoms such as diarrhea, flushing, sweating, tachycardia, and palpitation. These symptoms are usually associated with early DS. Whereas late DS is characterized by symptoms of hypoglycemia, which is resulted from hyperinsulinemia and occurs 1 to 3 h after carbohydrate ingestion [69, 79].

Screening for GDM in pregnant women post-bariatric surgery with oral glucose tolerance test may induce dumping syndrome. Therefore, other alternatives of blood glucose regular testing such as capillary glucose monitoring and continuous glucose monitoring are currently debatable to be used to substitute testing modalities [79].

## Effects of Bariatric Surgery on Fetal Outcomes

Despite the connection between post-RYGB pregnancy and decreased GDM, the risk of SGA and low birth weight might increase [64]. Furthermore, one study reported no effect on the neonates’ birth weight post-AGB and LSG while low birth weight was reported post-malabsorptive bariatric surgery (e.g., RYGB and LSG with duodenojejunal bypass) especially among mothers with anemia [13]. Recently, LSG has been performed more often than RYGB, indicating that more investigation is needed to study the effect of LSG on fetal and neonatal outcomes [39].

A prospective case–control study assessed 56 neonates of mothers who underwent RYGB and compared them to 56 neonates control groups of non-obese mothers. The study reported that 23% of the neonates from post-RYGB mothers were SGA compared to only 3.6% in the control group. Birth weight was lower in neonates from pregnancies post-RYGB (3.00 ± 0.57 kg) than in the control group (3.35 ± 0.43 kg), which was attributed to the calorie restriction as it plays a role in fetal development [11].

A study that evaluated 150 neonates born to mothers, who underwent three different types of bariatric surgeries, BPD, RYGB, and LSG, found the lowest average birth weight was among mothers post-BPD compared to the other two types of bariatric surgeries. However, a significantly lower average birth weight was found in the infants born post-maternal bariatric surgery than their siblings’ born pre-bariatric surgery [66].

A retrospective case–control study of 119 post-LSG pregnancies with a matched control group found higher SGA (14.3%) and low birth weight (12.6%) among the post-LSG group compared to the control group. Also, the study reported a lower rate of LGA (1.7%) and macrosomia (0.8%) than the control group with 19.3% and 7.6%, respectively [64]. Likewise, a Saudi study conducted on 145 post-LSG pregnancies found that 30.6% of the neonates were < 2.5 kg [10].

In light of the above, a study reported only 5 neonates (13.1%) had low birth weight out of 40 with an average weight of 3000.5 ± 572.2 kg. Only 8 were SGA (17.9%), and one had macrosomia from a mother who gained 30 kg during her pregnancy [16].

Moreover, a cross-sectional study evaluated the biochemical profile of 13 children who were born post-maternal bariatric surgery; the result shows 46.1% of the children had iron deficiency [80]. In a systematic review that evaluated complications for infants born after maternal bariatric surgery, those complications included NICU admission rates, and illness in the first 28 days of life, and rate of congenital malformations they found no significant differences [81].

### The Long-term Outcomes for Children Born Post-maternal Bariatric Surgery

A recent study concluded that neonates from mothers’ post-bariatric surgery had the lowest birth weight and higher SGA when compared to the other control groups (overweight/obese control and normal body weight control). On the other hand, the same group had a higher percent of body fat and waist circumference when compared to the control groups. They concluded that maternal bariatric surgery does not protect the neonates from the possible development of childhood obesity and overweight later in life [82].

Likewise, a qualitative study showed that maternal bariatric surgery does not protect children from obesity. Since the mother’s healthy lifestyle usually does not apply to the family, they consider it as individual change. Therefore, the majority of the children of this study were overweight and do not meet the recommended dietary and activity pediatric guidelines [83].

In a cross-sectional study that assesses nutritional status for children born after maternal bariatric surgery, the assessment found lower glucose levels throughout childhood among children who were exclusively breastfed. Also, lower fat mass was found among children who were both breastfeed and on artificial milk. This concluded the possible protecting role of breastfeeding from childhood obesity and its related comorbidities [80]. This corroborates the WHO guidelines to recommend exclusive breastfeeding for the first 6 months in an infant’s life [84].

## Conclusion

As the percentage of obesity continues to rise, there is an association between obesity in women of childbearing age and health conditions such as infertility and PCOS [3, 42]. In particular, the adverse health effect of obesity on pregnant women has been scientifically proven, as it resulted in pregnancy and neonatal complications such as GDM, PAHD, and macrosomia [6, 29]. However, little is known regarding the tendency of frequent unregulated pregnancies in Saudi and its potential in compromising the mothers’ health [22, 23].

Bariatric surgeries have proven to be safe and decrease the risk of some obesity-related complications in pregnancy [16, 47, 48]. In addition, studies have shown that timing between surgery and pregnancy is crucial for bariatric patients as the weight reduction is more significant in the first year post-surgery, affecting fetal growth [49, 50, 52]. The bariatric surgeons are aware of this issue; nonetheless, there is a growing need for reproductive health counseling preoperatively to avoid adverse effects that might arise during pregnancy post-bariatric surgery [46]. 

Moreover, post-bariatric females appear to have some knowledge about the importance of postponing pregnancy during the first year, in addition to nutritional knowledge about the dietary recommendation post-surgery [43, 58]. The fact that whether the patients are following the post-bariatric guidelines regarding preconception and dietary recommendations accurately needed to be addressed.

## Recommendations

Only a few studies address female patients’ pre and post-bariatric surgery with pregnancy outcomes, and a few articles have assessed their maternal and neonatal status. Further studies with different control groups of pregnant women with obesity and non-obese pregnant to compare possible obstetrics’ outcomes are needed.

The tendency of weight trajectory post-bariatric pregnancy is to gain less weight than normal pregnancy which might lead to inadequate GWG particularly in those who became pregnant in less than 12 months post-bariatric surgery and consequently might result in SGA and low birth weight. Following the IOM guidelines for GWG is essential. Further investigations are needed on those guidelines for post-bariatric surgery pregnancy.

Pregnancy post-bariatric surgery may be considered a high-risk pregnancy. Micronutrients’ serum concentrations of vitamins A, B12, K, D, calcium, folic acid, and iron should be assessed. Supplementations are vital to prevent possible micronutrients deficiency, such as vitamins B12, D, A, folate, thiamine, iron, and calcium.

The role of clinical dietitians is significant to emphasize regular clinic follow-up, monitor GWG, and increase compliance to prescribed supplements. In addition to prescribe oral supplements for pregnant women who experience inadequate GWG or malnutrition.

Moreover, since malabsorptive bariatric procedures (e.g., RYGB) are linked with more fetomaternal adverse outcomes, different bariatric surgeries should be assessed to provide an alternative procedure for women of childbearing age. Collectively, a summary of our recommendations for post-bariatric surgery pregnancy is shown in Table 8.

**Table 8:** Summary of the recommendations for post-bariatric surgery pregnancy

	Preconception	During pregnancy	Postpartum and breastfeeding
Contraception	• Reproductive health counseling pre-bariatric surgery • To avoid oral contraceptives, due to decrease the drug bioavailability post-bariatric surgery • To use long-acting reversible contraception (etonogestrel implants and intrauterine devices)		
Surgery-to-conception interval	• Postponing pregnancy from 12 to 18 months post-surgery • The dramatic weight loss occurs in the first year		
Nutritional intake	• Monitor the weight prior to pregnancy • In case of underweight to refer patient to clinical dietitian to correct the weight • If the pregnant is obese, it is preferable to lose weight before pregnancy to avoid obesity-related complications in pregnancy	• Monitor nutrition intake during pregnancy and assess for GWG if it is inadequate or excessive • To avoid excessive or inadequate gestational weight gain; appropriate gestational weight gain 11.5–16 kg for normal BMI as the IOM guidelines stated • Protein intake should be at least 60 g per day • Oral supplementation might be considered in case of inadequate nutrient intake or in the presence of hyperemesis gravidarum	• Ensure adequate calorie and protein during breastfeeding • Avoid excessive calories to avoid weight retention after pregnancy
Maternal and fetal screening		• Guidelines for pregnant women post-bariatric surgery should be considered as they are high-risk pregnancies as diabetic and hypertensive pregnancies • Check fasting glucose level and hgb A1C if there is a history of diabetes • Check fetal growth every 4–6 weeks of pregnancy starting from the 24th week for LGA and SGA • Oral glucose tolerance test at 24–28 weeks as possible. Noted that it was associated with dumping syndrome in some cases of post-bariatric surgery pregnancy	
Laboratory assessment	• Serum indices to be checked every 3 months: full blood count, vitamins A, B12, iron, ferritin, transferrin, and folic acid • Serum indices to be checked every 6 months: serum vitamin K1, vitamin D, protein, albumin, calcium, phosphate, magnesium, and PTH. In addition to renal and liver function Other extra serum indices to be checked especially during the 1st trimester: serum zinc, copper, selenium, and vitamin E	• Serum indices to be checked every 3 months: full blood count, vitamins A, B12, iron, ferritin, transferrin, and folic acid. In addition to transcobalamin • Serum indices to be checked every 6 months: INR, prothrombin time, serum vitamin K1, vitamin D, protein, albumin, calcium, phosphate, magnesium, and PTH. In addition to renal and liver function • Other extra serum indices to be checked especially during the 1st trimester: serum zinc, copper, selenium, and vitamin E	
Micronutrient’s supplementations	• Folic acid 0.4 mg should be taken daily since preconception and in the 1st trimester, 4–5 mg if obese or diabetic • Vitamin B12 taken as 1 mg IM for 3 months • Vitamin A taken as beta-carotene form. 5000 IU • If vitamin K deficiency noted to be taken orally in weekly doses • To keep vitamin D level above 50 nmol/L (1000 IU) • Add calcium as needed. 1200–1500 mg including dietary intake • Iron 45–60 mg (elemental iron) • Thiamine > 12 mg	• Thiamine supplementation 300 mg with vitamin B complex 3 times daily if pregnant women with vomiting. In case of prolonged vomiting, intravenous route should be considered • To continue with the suplmentation as periconception period • Additional supplements to be given in case of deficiencies	
